# Surface roughness rather than surface chemistry essentially affects insect adhesion

**DOI:** 10.3762/bjnano.7.139

**Published:** 2016-10-18

**Authors:** Matt W England, Tomoya Sato, Makoto Yagihashi, Atsushi Hozumi, Stanislav N Gorb, Elena V Gorb

**Affiliations:** 1 National Institute of Advanced Industrial Science and Technology (AIST), 2266-98, Anagahora, Shimoshidami, Moriyama, Nagoya 463-8560, Japan; 2 Nagoya Municipal Industrial Research Institute, 4-41, Rokuban, Atsuta, Nagoya 456-0058, Japan; 3 Zoological Institute: Functional Morphology and Biomechanics, Kiel University, Am Botanischen Garten 9, D - 24118 Kiel, Germany

**Keywords:** insect attachment, superhydrophilicity, superhydrophobicity, superoleophobicity, surface structures

## Abstract

The attachment ability of ladybird beetles *Coccinella septempunctata* was systematically investigated on eight types of surface, each with different chemical and topographical properties. The results of traction force tests clearly demonstrated that chemical surface properties, such as static/dynamic de-wettability of water and oil caused by specific chemical compositions, had no significant effect on the attachment of the beetles. Surface roughness was found to be the dominant factor, strongly affecting the attachment ability of the beetles.

## Introduction

The development of functional coatings that artificially mimic the properties of surfaces found in nature [1–4] to produce exceptional wetting/dewetting properties, such as superhydrophobicity, superhydrophilicity, and superoleophobicity (more commonly known as superamniphobicity or superomniphobicity), has been a major topic for research over the past decade [5–15].

There are countless examples of functional surfaces inspired by plants, such as lotus leaves [5,13] and the pitchers of carnivorous plants [9,14] that can be used to tune the wetting/de-wetting properties of surfaces on various substrates. Certain of these natural surfaces can effectively prevent wetting by water, while simultaneously protecting against attachment by insects by taking advantage of the same or very similar surface features [16–23]. Unfortunately, these natural anti-attachment properties have received relatively little attention from researchers working on surface science and engineering [24–25]. Another possible reason might be that the properties of unwettable biological surfaces, other than surface wetting/de-wetting, have not been tested. The question of whether surface chemistry or surface roughness is primarily responsible for natural anti-attachment properties has also not been fully resolved. Therefore, a comparative study of the attachment behavior of insects on artificially designed (low/high surface energy) surfaces of varying surface roughness, has been postulated as an effective strategy to identify the most important parameters influencing insect attachment.

Many insects, including beetles, can attach to inverted surfaces using specific hairy adhesive pads, covered with tenent setae, which secrete an adhesive fluid which typically consists of a mixture of alcohols, fatty acids, and hydrocarbons [26–32]. Several hypotheses exist on how plant surfaces prevent insect attachment. These are typically based on (1) the reduction of the contact area between the substrate and the insect adhesive pad through surface micro-roughness, (2) a decrease in substrate surface energy that enhances de-wetting of the insect attachment fluid, (3) fluid absorption by textured substrates, (4) contamination of the insect pads by easily erodible particles of the substrate, and (5) the reduction of wetting by pad fluid due to coverage of the substrate by another fluid (or solid which can be dissolved by the pad fluid) [22]. These mechanisms are to some extent conventional strategies utilized in functional surface design that gives us a unique chance to develop artificial surfaces with such properties, and test their anti-adhesive effects on insects [5–8,10–13,15]. However, recent studies on insect attachment have yielded contradicting results. For example, a previous experimental study on attachment of the beetle *Gastrophysa viridula* to the leaf surface of its host plant *Rumex obtusifolius*, and artificial micro-roughened and smooth (hydrophobic and hydrophilic) surfaces, has shown a stronger insect performance on smooth surfaces when compared to those with micro-roughness [33]. It was also found that surface hydrophobicity alone resulted in some decrease in the attachment force of the beetles, but when combined with surface micro-roughness it caused an even more pronounced reduction. Prüm et al. [17] measured the traction force of the beetle *Leptinotarsa decemlineata* on different plant surfaces and their artificial replicas, and reported that surface roughness exerted a strong influence on attachment, whereas surface chemistry was found to have no significant influence, despite both of these affecting the magnitude of water contact angles (CAs). Additionally, the attachment of the leaf beetle *Gastrophysa viridula* did not strongly depend on the free energy of the surface of the substrate [34]. More recently, the attachment strength of the beetle *Galerucella nymphaeae* on surfaces with different surface energies, showing CAs in the range from 6° to 109°, was examined [35]. These beetles, both at their larval and adult stages, showed the highest forces on surfaces with water CAs close to 83° (similar to those of their host plant), while hydrophilic (CAs of 6 and 26°) and hydrophobic (CA of 109°) surfaces caused a reduction of their adhesive ability.

A strong dependence of adhesive ability on the chemistry of the substrates during locomotion underwater was recently found for the beetle *Gastrophysa viridula* [34]*.* Using air bubbles trapped between their adhesive setae, these beetles are able to walk on flooded substrates, including those under water. Their attachment to hydrophilic surfaces was reduced when under water, compared to their attachment in air; whereas the attachment to hydrophobic surfaces under water was considerably stronger, and comparable to that observed in air. The oil-covered hairy pads on the feet of the beetle show a pinning effect, which retains air bubbles, and capillary attachment is produced by bubbles in contact with the hydrophobic substrate. Additionally, the liquid bridges of the pad between the foot and the substrate also produce capillary forces. Inspired by this idea, artificial silicone polymer structures with underwater adhesive properties were fabricated [34].

Thus, the relationship between surface structures and the attachment of insects, in combination with their particular chemical/physical properties, has not yet been fully resolved. Therefore, in order to obtain a deeper understanding of this bio-attachment phenomenon, it is crucial to systematically investigate the influence of both the surface chemistry and surface morphology on insect attachment properties using a greater range of surfaces with different surface wettabilities, in combination with both smooth and rough surface textures. Specifically, we focused our attention on both the static CAs of water and oil (*n*-hexadecane), and their dynamic (advancing (θ_A_) and receding (θ_R_)) CAs, especially CA hysteresis (Δθ, the difference between the values of θ_A_ and θ_R_), which is a mass-independent measure of the resistance to macroscopic liquid drop movement on inclined surfaces.

In this study, we investigated eight different surfaces. Three of these were smooth with different surface wettabilities, including two types of hydrophobic monolayers, with alkyl- and perfluoroalkyl-terminated functional groups. The third smooth surface was a hydrophobic/oleophilic alkylsilane-derived hybrid film, showing low CA hysteresis for water and *n*-hexadecane. In addition, we also studied three rough surfaces with different wettabilities. For these, we used a commercially available superhydrophobic coating system, which was also used to prepare a rough superhydrophilic surface, by subjecting it to vacuum UV (VUV) light treatment. A superomniphobic surface (defined here as a surface exhibiting both superhydrophobicity and superoleophobicity) was created using candle soot as templates for SiO_2_ nanoparticles, which were modified with a perfluoroalkylsilane monolayer. All surfaces were prepared on Si substrates. We measured traction forces of adult seven-spotted ladybird beetles *Coccinella septempunctata*, both males and females, on these six sample surfaces and two reference surfaces, i.e., smooth, hydrophilic silicon wafers (Si) and glass surfaces. Our sample surfaces displayed a wide range of surface chemical and topographical properties, and while both of these had a significant effect on the magnitude of CAs for probe liquids, the attachment abilities of the ladybird beetles were found to be predominantly influenced by the surface topography.

## Experimental

### Materials

Ethanol, 0.01 M HCl, and *n*-hexadecane were purchased from Wako Pure Chemical Industries Ltd. (Osaka, Japan). Tetramethoxysilane (TMOS, Si(OCH_3_)_4_) and *n*-octadecyltrimethoxysilane (ODS, CH_3_(CH_2_)_17_Si(OCH_3_)_3_) were purchased from Tokyo Chemical Industry Co., Ltd. (Tokyo, Japan). (Heptadecafluoro-1,1,2,2-tetrahydrodecyl)trimethoxysilane (FAS17, CF_3_(CF_2_)_7_CH_2_CH_2_Si(OCH_3_)_3_), (heptadecafluoro-1,1,2,2-tetrahydrodecyl)trichlorosilane (FAS17-Cl, CF_3_(CF_2_)_7_CH_2_CH_2_SiCl_3_), and decyltriethoxysilane (C_10_, CH_3_(CH_2_)_9_Si(OC_2_H_5_)_3_) were purchased from Gelest Inc. (Morrisville, PA, USA). Never Wet^TM^ (superhydrophobic coatings) was purchased from Rust-Oleum Corporation (Vernon Hills, IL, USA). All chemicals were used as received without further purification.

#### Preparation of flat and rough sample surfaces

Two smooth, hydrophobic monolayer-covered surfaces, terminated with octadecylsilyl (CH_3_(CH_2_)_17_-) or perfluoroalkyl (CF_3_(CF_2_)_7_CH_2_CH_2_-) groups, were prepared using chemical vapor deposition (CVD) of ODS or FAS17 [36], respectively. UV–ozone treated Si substrates (2 × 2 cm^2^ and 5 × 5 cm^2^) were placed on a heat-resistant glass plate (18 × 18 × 0.4 cm^3^) with a small aluminum-made vessel, or alternately placed in a Teflon container with a glass vessel, containing 0.2 mL of organosilane (ODS or FAS17), in a dry N_2_ atmosphere at less than 5 % relative humidity. Another heat-resistant glass plate was then placed on top of it using an O-ring (approximately 150 mm diameter and 8 mm thickness) as a spacer, and the four corners of the glass plates were secured using four clamps. Alternately, the Teflon container was sealed with an airtight screw-on Teflon cap. The reaction container was then heated for three days in an oven maintained at 180 °C for ODS and 150 °C for FAS17. Finally, the treated samples were rinsed with *n*-hexane, then water, and finally blown dry with a stream of N_2_. Besides these two flat monolayer-covered surfaces, we also prepared a smooth alkylsilane (C_10_)-derived hybrid film using conventional co-hydrolysis and co-condensation [10]. Briefly, precursor solutions were prepared by mixing C_10_ and TMOS in an ethanol/hydrochloric acid solution for 24 h at room temperature (25 ± 2 °C). The typical molar ratio of the precursor solution was 0.73 C_10_:2.92 TMOS:32 EtOH:14 H_2_O:7.7 × 10^−3^ HCl. The precursor solution was then spin-coated (500 rpm for 5 s and 1000 rpm for 10 s) onto UV–ozone-cleaned Si substrates (5 × 5 cm^2^) at room temperature, under a relative humidity of (40 ± 5)%. All samples were dried in air at room temperature for more than 24 h. Details of our preparation methods for the monolayers and hybrid film have been described elsewhere [10,12]. Three rough surfaces, each showing different wetting properties (superhydrophobicity, superhydrophilicity, and superomnipobicity) and morphologies were prepared as follows. The commercially available Never Wet coating system was used to prepare two rough superhydrophobic surfaces. Base coats were first deposited onto UV-cleaned Si substrates (2 × 2 cm^2^ and 5 × 5 cm^2^), then dried in air at room temperature (25 ± 2 °C) for more than 30 min. Next, topcoats were deposited onto the surfaces and cured at 100 °C for 24 h, hereafter referred to as Never Wet. Superhydrophilic surfaces were prepared by exposing superhydrophobic Never Wet surfaces to VUV light generated from an excimer lamp (Ushio Inc., UER20-172 V; λ = 172 nm and 10 mW/cm^2^) at 10^3^ Pa for 2 min, hereafter referred to as VUV-Never Wet. Superomniphobic surfaces were prepared according to a method modified from a report previously published by Deng et al [37]. The candle-soot-covered Si substrates (2 × 2 cm^2^ and 5 × 5 cm^2^) were exposed to VUV light at 10^3^ Pa for 30 min. The samples were then exposed to TMOS vapor for 4 h at 80 °C using the CVD method described previously. Next, the samples underwent thermal calcination in air for 3 h at 600 °C in order to remove any organic components, and thus obtain SiO_2_ nanostructures. After VUV irradiation at 10^3^ Pa for 30 min, the samples were finally exposed to a FAS17-Cl vapor at room temperature (25 ± 2 °C) for more than 3 h under reduced pressure, hereafter referred to as Soot-TMOS-FAS17Cl.

#### Characterization of sample surfaces

The thicknesses of the ODS and FAS17 monolayers were measured using ellipsometry (Philips, PZ2000). The thicknesses of the superhydrophobic (Never Wet), superhydrophilic (VUV-Never Wet), and superomniphobic films (Soot-TMOS-FAS17Cl) were estimated from cross-sectional images acquired by a scanning electron microscope (SEM, Phenom Pro Scanning Electron Microscope, Phenom World). The surface morphologies of the samples were either observed using the same SEM system or by atomic force microscope in a tapping mode (AFM, XE-100, Park Systems), with a Si probe (910M-NCHR; spring constant of 42 N/m and response frequency of 330 kHz, Park Systems). The surface roughness (root-mean square roughness, *R*_rms_) were estimated using two separate techniques due to the huge disparity in the size of surface textures on smooth and rough samples. Our five smooth samples (glass, Si, ODS, FAS17, and C_10_-hybrid) were estimated by AFM, while those of the three rough samples (Never Wet, VUV-Never Wet, and Soot-TMOS-FAS17Cl) were measured using a stylus profilometer (Surftest SJ-301, Mitutoyo Corp.).

Static and dynamic CA data (θ_S_ and θ_A_/θ_R_ values) for water and oil (*n*-hexadecane) were recorded using CA goniometers (model CA-V150, Kyowa Interface Science). The CA data reported here were determined by averaging values measured at 5–10 different points on each surface of the sample. All values for each sample were in a range of ±2°.

Surface chemical properties of the surfaces of the samples were studied by applying X-ray photoelectron spectroscopy (XPS). Spectra were obtained using a Physical Electronics Quantum 2000 spectrometer with 200 μm spot size and monochromatic Al Kα radiation (1486.68 eV). The X-ray source was operated at 50 W and 15 kV with the pass energy of the analyzer at 29.35 eV. The pressure in the analysis chamber was around 6 × 10^−9^ Pa during all measurements. Core-level signals were obtained at a photoelectron takeoff angle of 15° (surface-sensitive mode) with respect to the sample surface. The binding energy (BE) scales were referenced to 284.6 eV, as determined by the locations of the peak maxima of the C 1s spectra of a hydrocarbon (CH*_x_*). Surface compositions were determined by the corresponding core-level spectral area ratios, calculated using the relative sensitivity factor method. The relative error for all XPS data used to determine surface composition was estimated to be ±2%.

#### Insect force tests

Insect attachment ability was studied in traction experiments with tethered adult seven-spotted ladybird beetles *Coccinella septempunctata* (Coleoptera, Coccinellidae) by using a load cell force transducer (10 g capacity, Biopac Systems Ltd., Santa Barbara, CA, USA) as described by Gorb et al [23]. Insects were collected near Stohl (surroundings of Kiel, Germany). Forces generated by both males and females walking horizontally on six different surfaces (five smooth and three rough, each with different surface chemical and physical properties, (see above)) were measured. Obtained force–time curves were used to estimate the maximal traction force. Experiments were performed at 23 °C temperature and 26–29% relative humidity. We tested 10 male and 10 females and carried out 160 traction tests in total.

The experimental design included eight successive force tests with each insect individual: first on smooth, hydrophilic glass to check insect fitness, then on the other seven samples (Si, ODS, FAS 17, C_10_-hybrid, Never Wet, VUV-Never Wet, and Soot-TMOS-FAS17Cl) in a random order. In order to regain their superhydrophilic properties, Si and VUV-Never Wet samples, prior to the force tests, were plasma treated for 8 min with compressed air (pressure = 2.0 mbar) by applying a low pressure plasma system (Zepto, Diener electronic, Ebhausen, Germany), working at 100 W and 40 kHz regime. The samples were used in traction force experiments during maximum of 60 min after the plasma treatment.

Statistical analyses of the force values were carried out with SigmaStat 3.5 (Systat Software Inc., Point Richmond, CA, USA). For one-way ANOVA (Kruskal–Wallis one-way ANOVA on ranks), data obtained with males and females were pooled together. The effects of the sex of insect individuals and the surface type on the traction force values were examined using two-way ANOVA. Pairwise comparisons of sexes and surfaces were performed with the Tukey test. Force values are given as the mean ± standard deviation (SD).

## Results and Discussion

**Figure 1:**
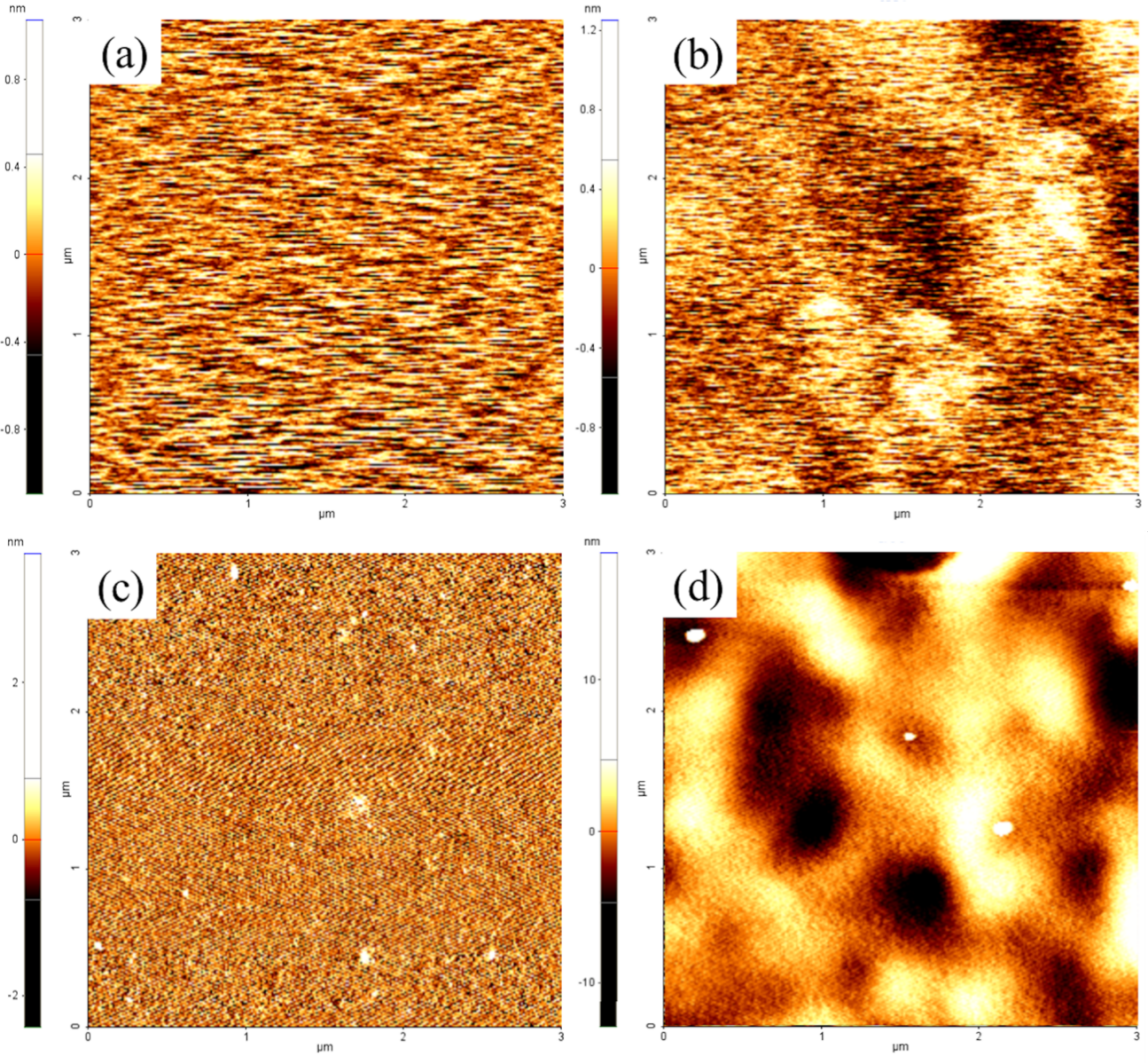
Typical AFM images of Si substrates before (a) and after (b) ODS-monolayer formation, (c) FAS17-monolayer formation, and (d) C_10_-hybrid film formation (scan areas are 3 × 3 μm^2^).

**Figure 2:**
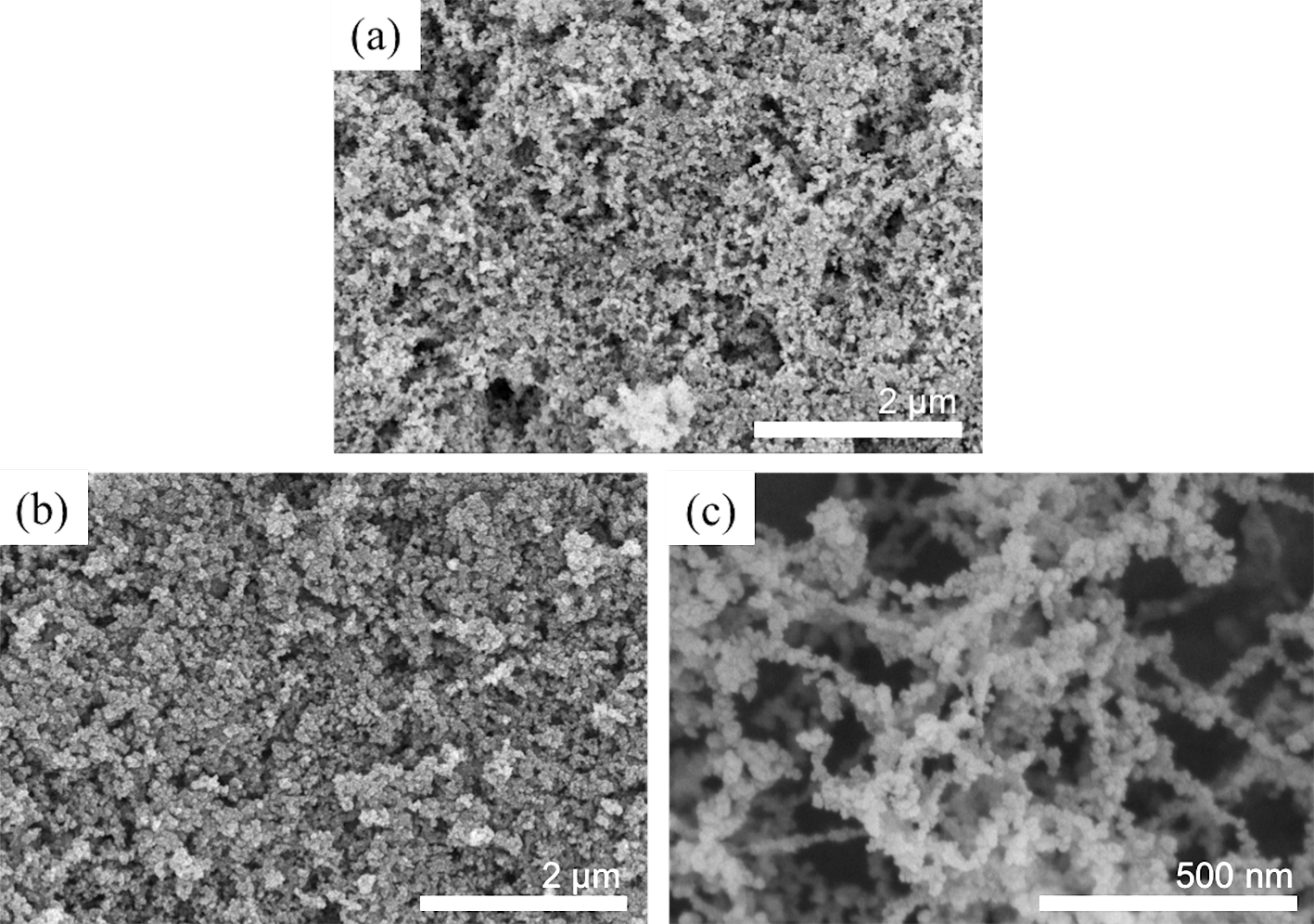
Typical top-down-view SEM images of sample surfaces: (a) Never Wet, (b) VUV-Never Wet, and (c) Soot-TMOS-FAS17Cl.

As shown in Figure 1, all sample surfaces observed by AFM appeared to be smooth over the entire scanning areas (3×3 μm^2^). The average *R*_rms_ values of ODS- and FAS17-monolayer-covered Si surfaces were very low (Table 1), indicating that the Si substrates could be modified uniformly (their thicknesses were between 1 and 2 nm) without any marked change in morphology (compared with the *R*_rms_ of an Si surface). Although the film thickness and *R*_rms_ of the C_10_-hybrid film itself was up to 700 times thicker (700 nm) and approximately 10 times higher (1.24 nm), respectively, than those of ODS and FAS17 monolayers, its surface was also fairly smooth. In contrast, SEM images shown in Figure 2 confirmed that films of the three rough surfaces, Never Wet, VUV-Never Wet, and Soot-TMOS-FAS17Cl, were substantially thicker (estimated by cross-sectional SEM images) than the monolayers and hybrid film, and also highly textured (Table 1). Due to these particulate film formation on these surfaces, their *R*_rms_ values were extremely high, around three to four orders of magnitude higher than those of the four smooth surfaces, as estimated by a stylus profilometer.

**Table 1:** Surface properties of the samples.

sample	θ_S_ water	θ_A_/θ_R_ water	θ_S_ *n*-hexadecane	θ_A_/θ_R_ *n*-hexadecane	thickness	*R*_rms_^a^

Si	—	—	—	—	—	0.16 nm
ODS	103°	106°/96°	10°	12°/5°	1.9 nm	0.23 nm
FAS17	113°	121°/108°	70°	73°/60°	1.1 nm	0.27 nm
C_10_-hybrid	111°	114°/106°	35°	36°/35°	700 nm	1.24 nm
Never Wet	155°	160°/158°	dissolved	—	≈35 μm	5.2 μm
VUV-Never Wet	—	—	dissolved	—	≈35 μm	6.3 μm
Soot-TMOS-FAS17Cl	163°	165°/160°	155°	161°/153°	<1.6 μm	0.2 μm

^a^*R*_rms_ values of Si with and without an ODS-monolayer, FAS17-monolayer, and C_10_-hybrid film were estimated using AFM images (3 × 3 μm^2^) shown in Figure 2; those of Never Wet, VUV-Never Wet and Soot-TMOS-FAS17Cl were estimated by a stylus profilometer.

Surface dewetting properties were investigated by measuring static/dynamic CAs (θ_S_, θ_A_, and θ_R_) of water and *n*-hexadecane droplets. As shown in Table 1, they were found to be strongly dependent on both surface chemical and topographical properties. Water CA measurements confirmed that the glass, Si and VUV-Never Wet surfaces were superhydrophilic with θ_S_ close to zero, while the other five surfaces exhibited either hydrophobic (ODS, FAS17 and C_10_-hybrid surfaces; water CAs greater than 100°) or superhydrophobic (Never Wet and Soot-TMOS-FAS17Cl surfaces; water CAs larger than 150°) properties. Water droplets on the latter surfaces moved very easily and would roll off at very low tilt angles of the substrate because of their extremely large CAs and low CA hysteresis (Δθ = 2–5°). On the other hand, while the static/dynamic CAs of smooth C_10_-hybrid surfaces were considerably lower than those of the superhydrophobic surfaces, the CA hysteresis of water on the hybrid film was also low (Δθ = 8°, the lowest of the smooth surfaces). Thus, contrasting with the smooth ODS and FAS17 monolayer surfaces, water droplets similarly slid off the C_10_-hybrid surfaces at low tilt angles, regardless of the magnitude of their static/dynamic CAs [10,36].

The CAs of *n*-hexadecane showed significantly greater variation. Monolayers of ODS and FAS17, and C_10_-hybrid film surfaces were all oleophilic with θ_S_ of 10°, 70°, and 35°, respectively. Among them, the C_10_-hybrid film surface in particular exhibited negligible CA hysteresis (Δθ = 1°), and excellent dynamic dewettability [10]. Unfortunately, the Never Wet surfaces, both before and after VUV irradiation, were dissolved by the *n*-hexadecane. However, the Soot-TMOS-FAS17Cl surface exhibited very high θ_S_ (155°) and low CA hysteresis (Δθ = 8°) for *n*-hexadecane, and thus *n*-hexadecane droplets on this surface could move, without pinning, more smoothly than they could on a smooth FAS17 surface (Δθ = 13°).

The relationships between surface wettability and chemical composition of our sample surfaces were established using XPS. The surface chemical compositions of each sample are summarized in Table 2. All non-perfluorinated sample surfaces were primarily composed of three elements (Si, oxygen (O), and carbon (C)). As expected, the Never Wet surface showed a decrease in C concentration (ca. 4 atom %) and an increase in the surface O concentration (ca. 6 atom %) following only 2 min VUV irradiation, in agreement with its marked increase in hydrophilicity (superhydrophilicity). Interestingly, the XPS analysis also revealed that the Never Wet surface had the lowest concentration of C (ca. 24 atom %) of any non-perfluorinated sample surface and showed excellent static/dynamic dewetting behavior. In addition, in spite of both perfluorinated sample surfaces (FAS17 and Soot-TMOS-FAS17Cl) showing very high surface fluorine concentrations (above 40 atom %), the former surface displayed inferior static/dynamic dewettability compared to that of the latter one. This clearly indicated that surface roughness, rather than C and F concentrations, had the strongest influence upon surface wettability.

**Table 2:** Sample surface compositions as estimated by XPS.

sample	Si (atom %)	O (atom %)	C (atom %)	F (atom %)

Si	60.1	39.9	—	—
ODS	27.7	41.5	30.8	—
FAS17	12.7	13.3	33.1	40.9
C_10_-hybrid	18.2	29.1	52.7	—
Never Wet	27.7	46.1	24.2	—
VUV-Never Wet	27.6	52.0	20.4	—
Soot-TMOS-FAS17Cl	9.6	15.6	26.7	46.2

**Figure 3:**
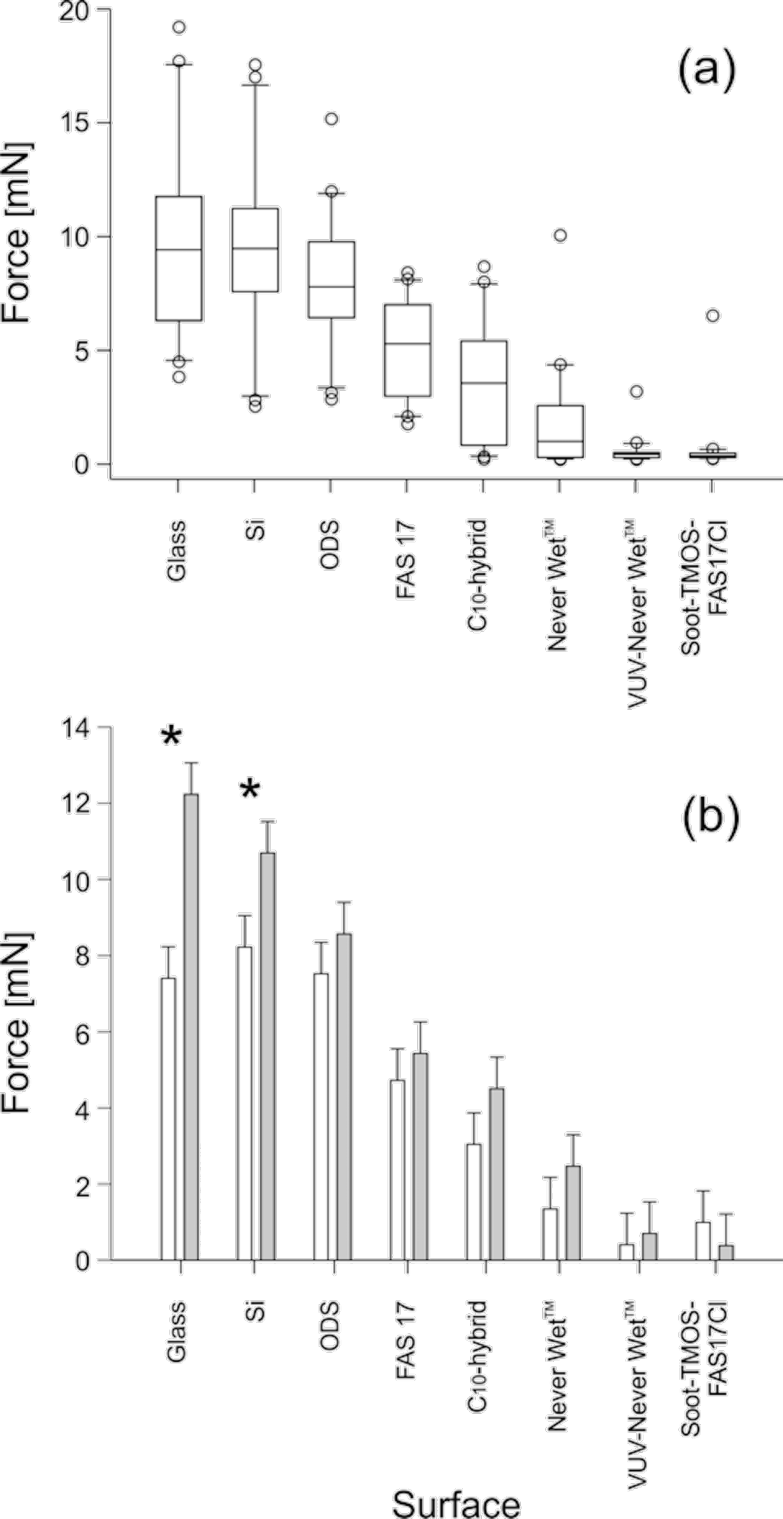
Traction forces of *Coccinella septempunctata* beetles measured on different sample surfaces: (a) data on males and females pooled together; (b) males (grey bars) and females (white bars). Asterisks in (b) indicate statistically significant differences between forces generated by males vs females (Tukey test, *P* < 0.05).

Based on the surface chemical and physical properties of our samples shown above, we next examined the attachment ability of *Coccinella septempunctata* beetles by measuring their traction forces on these surfaces. The average traction force produced by the insects on test surfaces ranged from 0.56 to 9.82 mN (Figure 3). All insects performed well on reference smooth, hydrophilic glass surfaces, showing force values either higher than (compared to C_10_-hybrid, Never Wet, VUV-Never Wet^TM^, and Soot-TMOS-FAS17Cl; Tukey test, *P* < 0.05) or similar to (compared to Si, ODS, and FAS17; Tukey test, *P* > 0.05) those obtained on other samples (Figure 3a). Among our samples (Kruskal–Wallis one-way ANOVA on ranks: *H*_6,139_ = 123.062, *P* < 0.001), two distinct groups can be clearly distinguished: smooth surfaces showing successful insect attachment (Si, ODS, and FAS17) and rough ones reducing the attachment (Never Wet, VUV-Never Wet, and Soot-TMOS-FAS17Cl). Interestingly, no statistical differences were detected between surfaces within each group (Tukey test, *P* > 0.05) indicating no significant effect of the surface dewetting properties on insect attachment. Only smooth C_10_-hybrid samples showed intermediate results, differing (*P* < 0.05) from smooth Si and similar surfaces (*P* > 0.05) to rough Never Wet (both Tukey test). Both the sex of the insect individuals (Figure 3b) and the surface type affected force values (two-way ANOVA: *F*_1,139_ = 4.992, *P* = 0.027 and *F*_6,139_ = 40.878, *P* < 0.001, respectively). However, there was no statistically significant interaction between these two factors (two-way ANOVA: *F*_6,139_ = 0.763, *P* = 0.601).

Our results show that the chemistry of smooth surfaces plays some role in the beetle attachment. The general trend is that hydrophobic and oleophobic substrates tend to reduce the attachment forces of beetles toward such surfaces. In addition, the results of previous studies on this matter have been fairly heterogeneous. In a recent publication, data from the literature dealing with force measurements of different insects on surfaces with different surface energies were carefully compared [35]. No significant dependence of insect attachment forces on water CAs was shown in the five experiments recorded in the four studies compared. However, seven experimental set-ups from six different studies revealed dependence of attachment forces on water CAs. Generally in the latter studies, at surfaces with water CAs above 100°, attachment forces were lower than those on surfaces with water CAs below 40°. It is important to note that in all of these studies different species, developmental stages, sexes and experimental designs were used. In some of these studies, insect species that are strongly specialized to host plants whose leaf surfaces have very specific surface energies (water CA about 80°), such as the beetle *Galerucella nympheae* which lives on the leaf surface of the water lily, the maximum attachment force was detected at the intermediate range of water CAs, approximately corresponding to those of the plant leaves [35].

**Figure 4:**
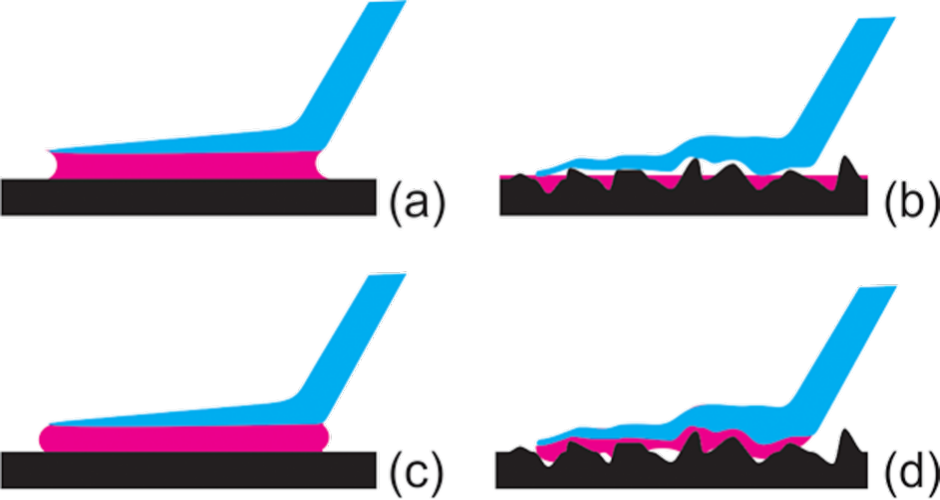
Hypothetical wetting behavior of the pad fluid (purple) in the contact region between the tenent setal tip (blue) and different sample surfaces (black): (a,c) flat surfaces, (b,d) textured surfaces; (a,b) fluids that readily wet the surfaces, (c,d) and fluids that poorly wet the surfaces.

Careful analysis of our data shows that there is no direct dependence of the attachment force of the beetle *Coccinella septempunctata* on the surface energies of the smooth substrates, as measured by the magnitude of CAs. It also seems that other factors, in addition to the surface energy, influence beetle attachment. These factors are presumably related to the different characters of chemical substances, which may mediate physical and chemical interactions in contact to different extents. In contrast, the fact that insect attachment is very sensitive to the substrate roughness is well-known, due to numerous previous studies on the subject [16,19–21,23,29,33,38]. Particularly strong reductions have been observed on textured substrates with *R*_rms_ in the range of 0.1 to 3 µm [16,23,29,33,38]. These results were also confirmed by the present study. However, in spite of our surfaces having completely different physical/chemical properties, there seem to be no differences in the attachment forces of beetles on rough substrates with different surface energies. We suspect this unusual attachment behavior is probably related to different physical parameters, particularly in the case of superhydrophilic/superoleophilic and superhydrophobic/superoleophobic surfaces. For the former types of surface, it is probably due to overly strong fluid absorption from the pads (Figure 4a,b). In the latter case, it is presumably due to wetting reduction by the pad fluid (Figure 4c,d). Additionally, at the sites of solid–solid contact between insect pads and substrates, the true contact area and contribution of van der Waals forces are believed to be effectively reduced for both types of textured substrate, despite them having different wettabilities (Figure 4b,d). In our present case, Never Wet and VUV-Never Wet samples would correspond to Figure 4b, while the Soot-TMOS-FAS17Cl surfaces are comparable to Figure 4d.

These effects were previously modeled to explain how the fluid flows on textured substrates with different roughness parameters and surface energies [39]. These numerical studies demonstrated that a higher density of geometrical surface structures of the rigid substrate results in a greater loss of fluid from the pad. The draining rate of the pad fluid is more rapid on fine roughness. A decreased affinity of the substrate to the pad fluid leads to significant reduction of the fluid loss. However, the substrate will not be wetted and will likely become slippery for the pad [39]. Our data demonstrated here provide clear experimental evidence for previous numerical predictions.

Attachment of male *C. septempunctata* was significantly stronger on the smooth hydrophilic surfaces. This effect was less pronounced or even vanished on our textured surfaces, which has been previously explained by the differences in the contact shape of the tenent setae [23,38].

In summary, we have clearly shown that the insect anti-adhesive effect is due to both surface chemistry and texture, but it is primarily driven by the substrate roughness, and less by surface chemistry. It seems to be a universal effect for both dry [40–41] and wet (but not glue-mediated) [23,29,38,42] adhesive systems.
